# A pyroptosis-related gene signature that predicts immune infiltration and prognosis in colon cancer

**DOI:** 10.3389/fonc.2023.1173181

**Published:** 2023-07-12

**Authors:** Mingjian Wu, Shuai Hao, Xiaoxiang Wang, Shuguang Su, Siyuan Du, Sitong Zhou, Ronghua Yang, Hanpeng Du

**Affiliations:** ^1^ Department of Gastrointestinal Surgery, Panyu Maternal and Child Care Service Centre of Guangzhou (He Xian Memorial Affiliated Hospital of Southern Medical University), Guangzhou, China; ^2^ Department of General Surgery, Jinling Hospital, Medical School of Nanjing University, Nanjing, China; ^3^ The First Clinical Medical College, Guangdong Medical University, Zhanjiang, Zhanjiang, Guangdong, China; ^4^ Department of Pathology, Panyu Maternal and Child Care Service Centre of Guangzhou (He Xian Memorial Affiliated Hospital of Southern Medical University), Guangzhou, China; ^5^ Department of Dermatology, The First People’s Hospital of Foshan, Foshan, Guangdong, China; ^6^ Department of Burn and Plastic Surgery, Guangzhou First People’s Hospital, South China University of Technology, Guangzhou, Guangdong, China

**Keywords:** colon cancer, pyroptosis, tumor immune microenvironment, prognosis, risk signature

## Abstract

**Background:**

Colon cancer (CC) is a highly heterogeneous malignancy associated with high morbidity and mortality. Pyroptosis is a type of programmed cell death characterized by an inflammatory response that can affect the tumor immune microenvironment and has potential prognostic and therapeutic value. The aim of this study was to evaluate the association between pyroptosis-related gene (PRG) expression and CC.

**Methods:**

Based on the expression profiles of PRGs, we classified CC samples from The Cancer Gene Atlas and Gene Expression Omnibus databases into different clusters by unsupervised clustering analysis. The best prognostic signature was screened and established using least absolute shrinkage and selection operator (LASSO) and multivariate COX regression analyses. Subsequently, a nomogram was established based on multivariate COX regression analysis. Next, gene set enrichment analysis (GSEA) and gene set variation analysis (GSVA) were performed to explore the potential molecular mechanisms between the high- and low-risk groups and to explore the differences in clinicopathological characteristics, gene mutation characteristics, abundance of infiltrating immune cells, and immune microenvironment between the two groups. We also evaluated the association between common immune checkpoints and drug sensitivity using risk scores. The immunohistochemistry staining was utilized to confirm the expression of the selected genes in the prognostic model in CC.

**Results:**

The 1163 CC samples were divided into two clusters (clusters A and B) based on the expression profiles of the 33 PRGs. Genes with prognostic value were screened from the DEGs between the two clusters, and an eight PRGs prognostic model was constructed. GSEA and GSVA of the high- and low-risk groups revealed that they were mainly enriched in inflammatory response-related pathways. Compared to those in the low-risk group, patients in the high-risk group had worse overall survival, an immunosuppressive microenvironment, and worse sensitivity to immunotherapy and drug treatment.

**Conclusion:**

Our findings provide a foundation for future research targeting pyroptosis and new insights into prognosis and immunotherapy from the perspective of pyroptosis in CC.

## 1 Introduction

Colon cancer (CC) has become one of the most common gastrointestinal malignancies worldwide, with approximately 1.15 million new cases in 2020, and is the fifth most common cause of cancer-related deaths (1). Despite the availability of comprehensive drugs, outcomes remain unsatisfactory, with a 40–60% five-year survival rate, which is a serious threat to human health (2). Therefore, an in-depth understanding of the mechanisms of CC development and effective assessment of prognostic differences among individuals are essential to achieve individualized and precise treatment and improve patient survival.

The most widely used prognostic staging system is the TNM staging system (3, 4), which also serves as a benchmark for establishing clinical treatment protocols for patients with CC. In addition, the assessment of microsatellite instability (MSI) status and KRAS or BRAF mutation status, also has prognostic value for patients with CC (5). With the advancement of genetic testing technology, it has been realized that tumors belong to a class of highly heterogeneous and complex diseases, and personalized prognostic analysis needs to be performed for different patients’ genomic characteristics. Because single-gene/factor prediction models have low accuracy, more studies have explored the value of polygene-based models in identifying novel immunotherapy targets and predicting cancer prognosis (6, 7).

Pyroptosis, also known as inflammatory cell necrosis, was first described in 2001 when it was recognized as a completely different mode of cell death than traditional apoptosis (8). Pyroptosis is a programmed cell death mediated by the gasdermin family of proteins (9), which manifests as continuous cell swelling until the cell membrane ruptures, leading to the release of cellular contents, which in turn activates an intense inflammatory response. In recent years, the role of pyroptosis in tumor pathogenesis has become increasingly prominent, and molecules of the pyroptosis signaling pathway and various inflammatory mediators released during pyroptosis have been found to be closely related to tumorigenesis, tumor development, and antitumor immunity (10). Although several studies have reported the relationship between pyroptosis and CC (11, 12), the mechanism by which pyroptosis affects the immune microenvironment through pro-inflammatory factors, thus influencing the prognosis and treatment response of patients with CC, requires further investigation.

Using The Cancer Genome Atlas (TCGA) and the Gene Expression Omnibus (GEO) databases, we aimed to explore the function of pyroptosis in CC and hoped to develop a pyroptosis-related gene signature to predict the prognosis and guide therapy for patients with CC that can provide more information for clinical treatment. Immune infiltration analysis was also performed to examine the influence of pyroptosis-related genes (PRGs) on regulation of the immune microenvironment. Finally, we explored the association between the risk scores and immunotherapy sensitivity, which could help identify new therapeutic targets.

## 2 Materials and methods

### 2.1 Data collection and processing

The CC dataset TCGA-COAD (n = 521) was downloaded from TCGA database (https://portal.gdc.cancer.gov) using the TCGAbiolinks package (13) of R software (version 4.1.1, http://r-project.org/), selecting the data type in TPM format. The clinical information of TCGA-COAD matched patients (n = 454), including age, survival status, follow-up time, and stage, was also downloaded and obtained using the GDC software. Data with no survival information or incomplete TNM staging information were excluded. In addition, “Masked Somatic Mutation” data in the TCGA database were selected using the TCGAbiolinks package as the somatic mutation data of patients with CC and were visualized using the maftools package (14).

To analyze the copy number variation (CNV) of key genes in the TCGA-COAD dataset, we downloaded the “Masked Copy Number Segment” data of patients (n = 976) using the TCGAbiolinks package and performed genomic identification of significant targets in cancer (GISTIC) 2.0 (15) analysis on the downloaded CNV fragment data using GenePattern (https://cloud.genepattern.org) to investigate the CNV of COAD, including the chromosomal arm level CNVs and the least common region between samples.

The sample-sourced reliable CC expression profiling datasets GSE17536 (16) and GSE39582 (17) were downloaded from the GEO (https://www.ncbi.nlm.nih.gov/geo/) database using the R package GEOquery (18). All samples in the datasets were derived from *Homo sapiens*, and the platforms were based on the GPL570 [HG-U133_Plus_2] Affymetrix Human Genome U133 Plus 2.0 Array. The GSE17536 dataset included 177 CC samples, and the GSE39582 dataset included 566 patients with CC and 19 healthy individuals’ colon tissue samples, among which 10 CC samples were excluded owing to the lack of survival information. Thus, a total of 733 CC samples from the GEO database were included in this study. The raw data of the GSE17536 and GSE39582 datasets were read using the GEOquery package, background corrected, and data normalized to obtain gene expression matrices. Batch effects were removed using the sva package (19) to obtain combined TCGA and GEO gene expression matrices, and the correction effect was demonstrated by plotting a boxplot (**Figure S1**).

### 2.2 Consensus clustering of pyroptosis-related genes

In this study, a set of PRGs was obtained from previously published literature (20), with a total of 33 genes, and pyroptosis genes that were significant for the TCGA+GEO dataset were selected. Unsupervised cluster analysis of these genes and the TCGA+GEO dataset was performed using the R package ConsensusClusterPlus (21). The number of clusters was set between 2 and 10, and 80% of the total sample was drawn in 1000 repetitions with the parameters clusterAlg = “km” and distance = “euclidean”.

### 2.3 Identification and analysis of differentially expressed genes

CC samples from the TCGA+GEO dataset were classified into two subtypes, clusters A and B, based on PRG clustering, and the differentially expressed genes (DEGs) in the gene expression matrix were screened using the limma package (22). DEGs were shown as volcano plots and heatmaps using the ggplot2 (23) and pheatmap (https://cran.r-project.org/web/packages/pheatmap/index.html/) packages, respectively. Log fold-change (logFC) absolute values > 1 and a P-value < 0.05 were set as the thresholds for DEGs. Upregulated DEGs in cluster B generally had values of logFC > 1 and a P-value < 0.05, whereas downregulated DEGs in cluster B typically had values of logFC < -1 and a P-value < 0.05.

### 2.4 Enrichment analysis of differentially expressed genes

Gene Ontology (GO) (24) analysis is a common approach for large-scale functional enrichment analysis, including biological process, molecular function, and cellular component. The Kyoto Encyclopedia of Genes and Genomes (KEGG) (25) is a widely used database that stores information on genomes, biological pathways, diseases, and drugs. Using the clusterProfiler package (26) for GO annotation and KEGG pathway enrichment analyses of pyroptosis gene regulators, a critical value of false discovery rate (FDR) < 0.05 was considered statistically significant.

To investigate the differences in biological processes between the different subgroups, we performed gene set enrichment analysis (GSEA) (27) based on the expression profiles of the high- and low-risk group datasets of CC samples from the TCGA+GEO dataset. GSEA is a computational method used to analyze whether a particular gene set is statistically different between two biological states and is commonly used to estimate changes in pathway and biological process activity. The gene set “c2.cp.kegg.v7.5.1. entrez.gmt” was downloaded from the MsigDB database (28) for GSEA analysis, and a FDR < 0.25 and P < 0.05 was considered significantly enriched. The gene set “c6.all.v7.5.1. symbols.gmt” was also downloaded from the MsigDB database, and gene set variation analysis (GSVA) was performed on the gene expression matrix using the GSVA package (29), with P < 0.05 indicating a significantly enriched oncogenic-related pathway.

### 2.5 Prognostic gene clustering and model construction

We used the TCGA+GEO dataset as a basis for evaluating the association between each DEG and overall survival (OS) using univariate COX proportional regression analysis and retained genes with P-values < 0.05, that is, PRGs. Unsupervised clustering analysis was performed on these genes and the TCGA+GEO dataset using the ConsensusClusterPlus package (21). The number of clusters was set between 2 and 10, and 80% of the total sample was drawn in 1000 repetitions with the parameters clusterAlg = “km” and distance = “euclidean”.

Least absolute shrinkage and selection operator (LASSO) regression (30) is a machine learning algorithm commonly used to construct diagnostic models that performs regularization to prevent overfitting and improve the accuracy of the model. Therefore, using the glmnet package (31), we applied the LASSO algorithm to eliminate multicollinearity and screen for meaningful variables in the univariate COX regression analysis, with parameters set.seed (2), family = “ COX”. To obtain more accurate independent prognostic factors (prognostic trait genes), we utilized multivariate COX regression analysis and performed final screening by stepwise regression. Finally, by considering the expression of the optimized genes and the associated estimated COX regression coefficients, the risk score was calculated using the “predict” function.


$$h_{0}\left(t,X\right)=h_{0}\left(t\right)*\text{exp}\left(\beta_{1\;}X_{1}+\beta_{2}X_{2}+\ldots +\beta_{n}X_{n}\right)$$

The regression coefficient, β, was utilized to obtain the hazard ratio by taking natural logarithm of exp(-*β*). *h*_0_ (t) is the baseline risk function; *h*(t,X) is the risk function associated with X (covariate) at time t. The value of the risk score calculated by the predict function is *h*(*t,X*). And the patients were divided into high risk and low risk groups according to the given risk scores. Patients were divided into high- and low-risk groups according to their risk scores. Kaplan–Meier analysis and log-rank test were applied to analyze the OS using the survival package. In addition, a nomogram was constructed using COX regression, and time-dependent subject operating characteristic (ROC) curves were used to assess survival prediction. The area under the ROC curve (AUC) values were calculated using the timeROC package (32) to measure prediction accuracy and correct curves to assess stability.

### 2.6 Gene mutation and CNV analysis

The maftools package (14) showed mutations in high- and low-risk groups and allowed the estimation of the tumor mutation burden (TMB) using the total number of non-synonymous mutations per megabase. GISTIC 2.03 (https://cloud.genepattern.org) was used to determine copy number changes to classify amplified or deleted genes.

### 2.7 Immune infiltration analysis

CIBERSORT (33) is based on the principle of linear support vector regression to deconvolute the transcriptome expression matrix and estimate the composition and abundance of immune cells in a mixture of cells. We uploaded the TCGA+GEO gene expression matrix data to CIBERSORTx (https://cibersortx.stanford.edu), combined with the LM22 signature matrix, and filtered the output for samples with P < 0.05 to derive the immune cell infiltration matrix. Histograms were plotted using the ggplot2 package to show the distribution of the 22 immune cell infiltrates in each sample. Heat maps were plotted to visualize the correlation between 22 immune cell infiltrates using the corrplot package (https://github.com/taiyun/corrplot). Correlation plots between immune cells and different subgroups were generated using the ggpubr package.

### 2.8 Immunotherapy and drug sensitivity analysis

Cellular features of immune infiltration determine immunophenotype and tumor escape mechanisms; we used the immunophenoscore (IPS) from The Cancer Immunome Atlas (34) (TCIA, https://tcia.at/home) to predict CTLA-4 and anti-PD-1 antibody responses. Based on the results of TCIA analysis, we compared the differences in CTLA4 and PD-1 expression between the high- and low-risk groups.

The Genomics of Drug Sensitivity in Cancer (GDSC) database (www.cancerrxgene.org/) (35) can be used to search oncology drug response data and genomic sensitivity markers. We used the pRRophetic algorithm (36) to construct ridge regression models based on gene expression profiles to predict the sensitivity of high- versus low-risk groups to common anticancer drugs based on IC50 values.

The tumor immune dysfunction and exclusion (TIDE) (37) score (http://tide.dfci.harvard.edu) can predict potential tumor treatment response to ICB, a computational algorithm based on gene expression profiles. Based on the results of the TIDE analysis, we compared the high- and low-risk groups with differences in various indicators of tumor immunotherapy.

### 2.9 Patients tissue specimens and immunohistochemistry (IHC) staining

A total of 25 patients fulfilling the inclusion criteria (Histologically confirmed stage II or III or IV colon cancer) at Panyu Maternal and Child Care Service Centre of Guangzhou (He Xian Memorial Affiliated Hospital of Southern Medical University) between 2020 and 2022, were included in the present study.

The detailed of IHC procedure and histologic scoring and analysis were performed as described (38). Briefly, specimens were incubated with individual primary antibodies (MMP3, Abcam; CXCL2, SAB Signalway Antibody; MMP12, SAB Signalway Antibody; KRT23, SAB Signalway Antibody; TNFAIP6, SAB Signalway Antibody; CCL8, Solarbio) and then washed and incubated with HRP–conjugated secondary antibody (goat anti-rabbit, 1:500, Cell Signaling Technology). Colorimetric reaction was with DAB.

### 2.10 Statistical analysis

All data calculations and statistical analyses were performed using R software (https://www.r-project.org/, v4.1.1). For comparisons of continuous variables between the two groups, an independent *t*-test was used to estimate normally distributed variables, and the Mann–Whitney U test was used to analyze non-normally distributed variables. All P-values were two-sided, and statistical significance was set at P < 0.05.

## 3 Results

### 3.1 Data pre-processing

**Figure 1:**
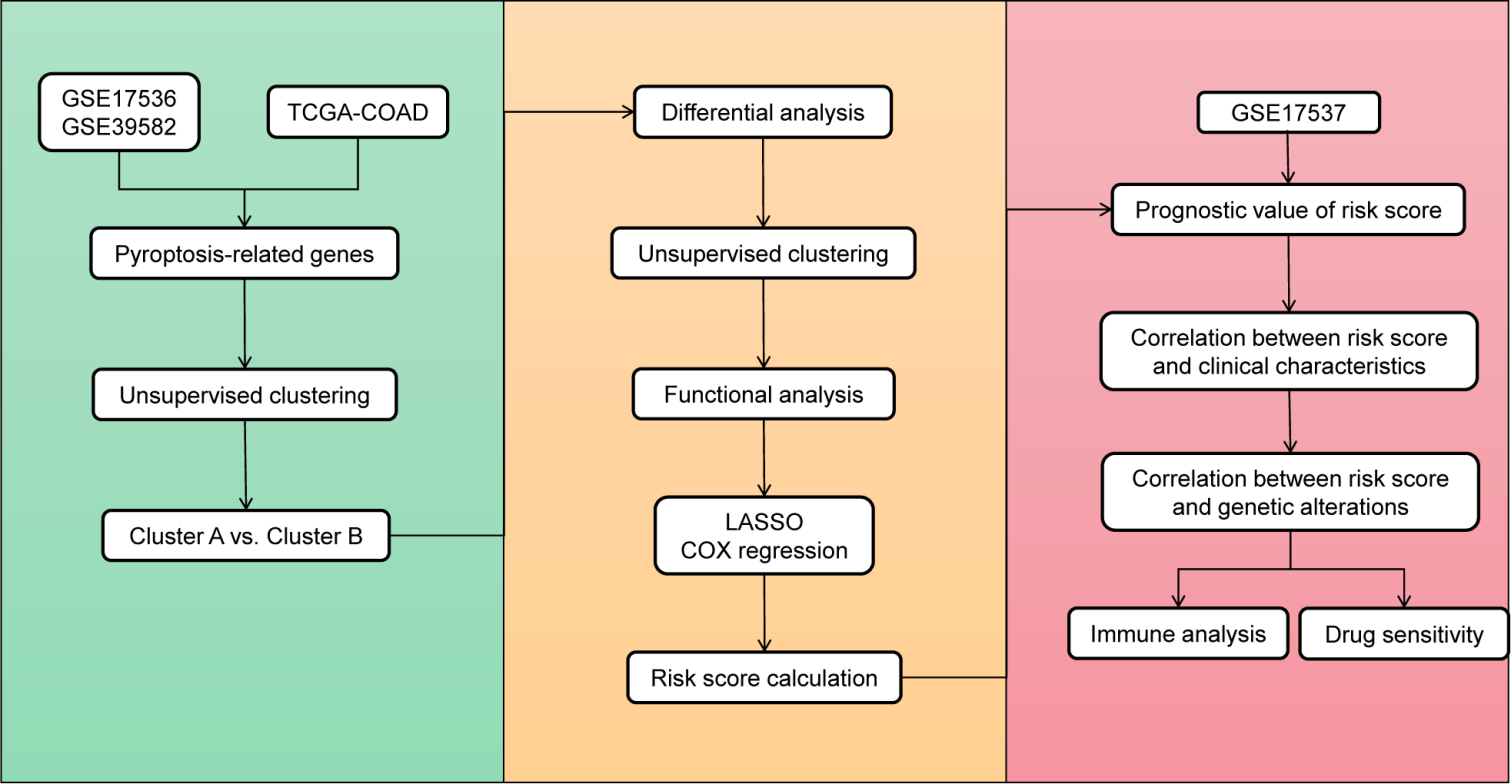
Overview of the study flowchart.

**Figure 1** shows an overview of the study flowchart. The gene expression matrices from TCGA-COAD, GSE17536, and GSE39582 datasets underwent standardization, preprocessing, and batch effect removal. **Figures S1A, B** presents the boxplot graph of the dataset before and after batch effect removal. Principal component analysis (PCA) was then conducted on the merged dataset to evaluate the impact of batch effect removal (**Figures S1C, D**). The result showed that before batch effect removal, the distance between GSE17536, GSE39582, and TCGA-COAD datasets were relatively far apart. However, after batch effect removal, the three datasets were evenly distributed together, indicating that the batch effect in the merged dataset samples was significantly reduced and the dataset can be used for subsequent analysis.

### 3.2 Characterization of pyroptosis subgroups in colon cancer

**Figure 2:**
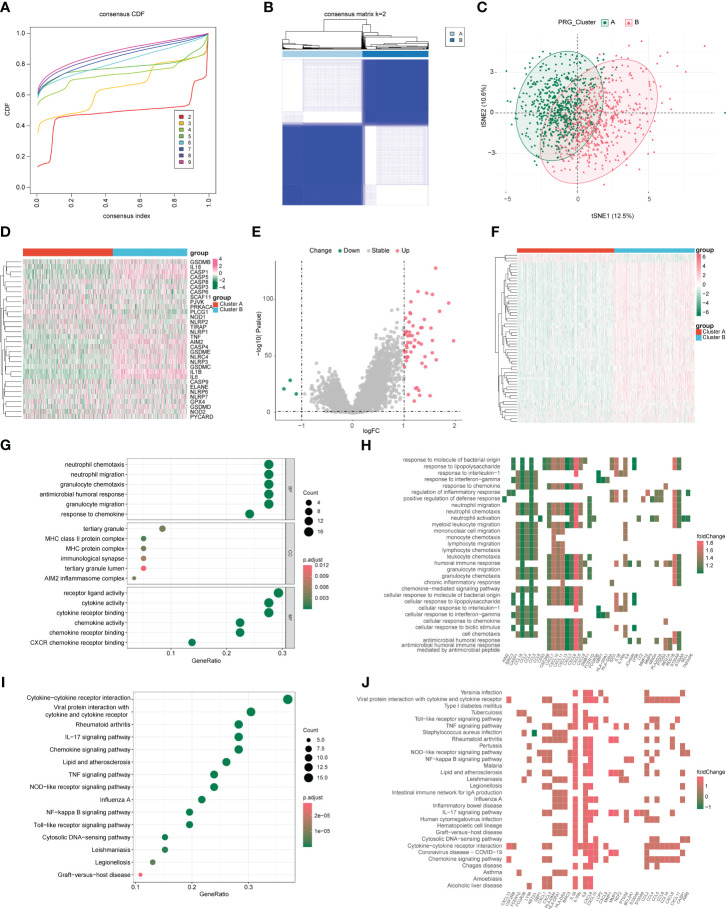
Characterization of pyroptosis subgroups in colon cancer and screening of subtype-associated genes. **(A)** CDF plot when k takes different values. **(B)** Heat map of sample clustering at k = 2. **(C)** Principal component analysis (PCA) of two pyroptosis subgroups. **(D)** Expression of pyroptosis genes in different subgroups. **(E)** Volcano plot of differentially expressed genes; red and green indicate upregulated and downregulated genes, respectively, in the cluster 2 group. **(F)** Differentially expressed genes in heat map; red and green indicate high and low expression, respectively. **(G, H)** Gene Ontology (GO) analysis of bubble and heat maps. **(I, J)** Kyoto Encyclopedia of Genes and Genomes (KEGG) analysis of bubble and heat maps.

Based on the expression of pyroptosis genes, unsupervised clustering analysis was used, and the clustering results are shown in **Figure 2A**. We chose the clustering results at k = 2 (**Figure 2B**), which were divided into two pyroptosis subgroups: cluster A (n = 637) and cluster B (n = 526). Based on sample expression profiles, we distinguished the distribution characteristics of the different subgroups using principal component analysis (PCA) and found a clear distinction (**Figure 2C**). We then compared the expression of PRGs between the two subgroups and found significant differences in PRG expression (**Figure 2D**).

To assess the transcriptomic differences between pyroptosis regulatory patterns, we performed differential analysis of different subgroups (clusters A and B) and obtained 59 DEGs as pyroptosis-related signature genes, of which 56 were upregulated and 3 were downregulated in cluster B (**Figure 2E, F**). We performed GO/KEGG analysis on the differential genes with a P-value cutoff of 0.05 and obtained the results shown in **Figures 2G–J**, **S2**, and **Table 1**. GO analysis shows that pyroptosis gene regulators were closely related to biological processes such as receptor ligand activity, neutrophil chemotaxis, neutrophil migration, granulocyte chemotaxis, and cytokine receptor binding. KEGG analysis shows that pyroptosis gene regulators affected viral protein interactions with cytokines and cytokine receptors, rheumatoid arthritis, IL-17 signaling pathway, cytokine-cytokine receptor interaction, and chemokine signaling pathway.

**Table 1:** GO/KEGG enrichment analysis.

ONTOLOGY	ID	Description	pvalue	p.adjust	qvalue
BP	GO:0030593	neutrophil chemotaxis	1.23E-23	2.15E-20	1.28E-20
MF	GO:0008009	chemokine activity	1.80E-22	3.93E-20	1.80E-20
BP	GO:1990266	neutrophil migration	2.48E-22	1.66E-19	9.91E-20
BP	GO:0071621	granulocyte chemotaxis	2.84E-22	1.66E-19	9.91E-20
BP	GO:0019730	antimicrobial humoral response	2.73E-21	1.20E-18	7.15E-19
BP	GO:0097530	granulocyte migration	5.43E-21	1.90E-18	1.14E-18
MF	GO:0042379	chemokine receptor binding	2.53E-20	2.76E-18	1.27E-18
BP	GO:1990868	response to chemokine	2.87E-20	7.19E-18	4.30E-18
BP	GO:1990869	cellular response to chemokine	2.87E-20	7.19E-18	4.30E-18
BP	GO:0097529	myeloid leukocyte migration	8.11E-20	1.78E-17	1.06E-17
MF	GO:0005125	cytokine activity	1.57E-17	1.14E-15	5.24E-16
MF	GO:0005126	cytokine receptor binding	1.44E-16	7.84E-15	3.60E-15
MF	GO:0045236	CXCR chemokine receptor binding	2.59E-16	1.13E-14	5.17E-15
MF	GO:0048018	receptor ligand activity	8.64E-14	3.14E-12	1.44E-12
MF	GO:0030546	signaling receptor activator activity	1.05E-13	3.28E-12	1.51E-12
MF	GO:0001664	G protein-coupled receptor binding	4.73E-12	1.29E-10	5.92E-11
CC	GO:0042613	MHC class II protein complex	1.43E-05	0.001756791	0.000992282
CC	GO:0042611	MHC protein complex	5.75E-05	0.003538542	0.001998663
CC	GO:0097169	AIM2 inflammasome complex	0.000133675	0.004203185	0.002374071
CC	GO:0070820	tertiary granule	0.000136689	0.004203185	0.002374071
CC	GO:0001772	immunological synapse	0.000257665	0.006338555	0.003580185
CC	GO:1904724	tertiary granule lumen	0.000615378	0.012048803	0.006805486
KEGG	hsa04061	Viral protein interaction with cytokine and cytokine receptor	1.19E-16	1.51E-14	8.28E-15
KEGG	hsa05323	Rheumatoid arthritis	1.75E-15	8.58E-14	4.69E-14
KEGG	hsa04657	IL-17 signaling pathway	2.03E-15	8.58E-14	4.69E-14
KEGG	hsa04060	Cytokine-cytokine receptor interaction	1.35E-13	4.28E-12	2.34E-12
KEGG	hsa04668	TNF signaling pathway	1.79E-11	4.54E-10	2.49E-10
KEGG	hsa04062	Chemokine signaling pathway	2.36E-11	5.00E-10	2.74E-10
KEGG	hsa05417	Lipid and atherosclerosis	1.48E-09	2.68E-08	1.47E-08
KEGG	hsa04621	NOD-like receptor signaling pathway	3.86E-09	5.98E-08	3.27E-08
KEGG	hsa04064	NF-kappa B signaling pathway	4.71E-09	5.98E-08	3.27E-08
KEGG	hsa04620	Toll-like receptor signaling pathway	4.71E-09	5.98E-08	3.27E-08
KEGG	hsa05164	Influenza A	2.70E-08	3.12E-07	1.70E-07
KEGG	hsa04623	Cytosolic DNA-sensing pathway	4.95E-08	5.24E-07	2.87E-07
KEGG	hsa05140	Leishmaniasis	2.03E-07	1.98E-06	1.08E-06
KEGG	hsa05134	Legionellosis	6.75E-07	6.12E-06	3.35E-06
KEGG	hsa05332	Graft-versus-host disease	3.34E-06	2.82E-05	1.54E-05
KEGG	hsa05133	Pertussis	3.75E-06	2.98E-05	1.63E-05

### 3.3 Characterization of differential gene subgroups in colon cancer

**Figure 3:**
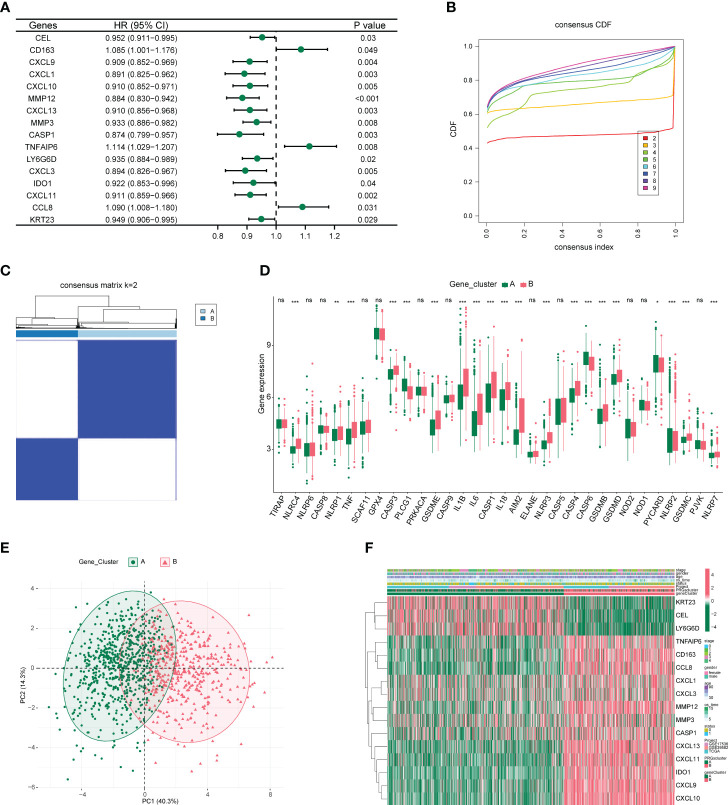
Characterization of differentially expressed gene subgroups in colon cancer. **(A)** Forest plot of univariate COX proportional regression analysis. **(B)** CDF plot when k takes different values. **(C)** Heat map of samples clustering at k = 2. **(D)** Box plot of differential expression of pyroptosis-related genes among different subgroups. *P<0.05, **P<0.01, ***P<0.001. **(E)** Principal component analysis (PCA) of two differentially expressed gene subgroups. **(F)** Heat map of differential expression of 16 genes between different subgroups.

From 59 DEGs, we selected 16 DEGs (P-value < 0.05) using univariate COX proportional regression analysis (**Figure 3A**) and then used unsupervised clustering to identify different DEG subgroups. We concluded that the differences between these subgroups better reflected the characteristics of the pyroptosis subgroup. When k = 2, the best grouping was found (**Figures 3B, C**), and the distribution characteristics of different DEG subgroups were evaluated using PCA and a clear distinction was found between the two subgroups (**Figure 3E**). We then performed differential analysis and mapped the differential expression of PRGs and 16 DEGs between the different DEG subgroups (**Figures 3D, F**).

### 3.4 Construction of prognostic models

**Figure 4:**
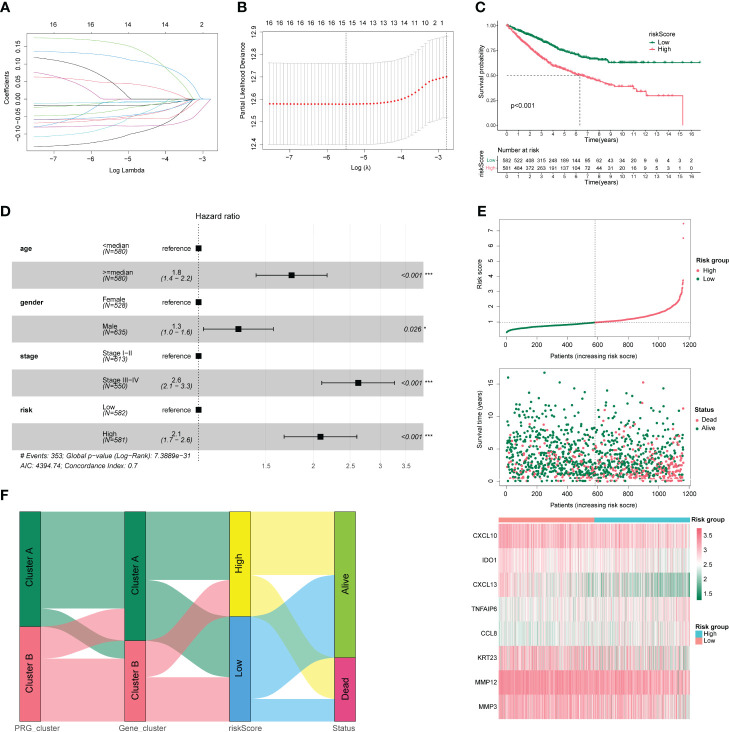
Clinical characteristics of colon cancer. **(A)** Number and coefficients of enrolled characteristics at different λ states during least absolute shrinkage and selection operator (LASSO) model building. **(B)** Optimal λ values for LASSO model. **(C)** Survival curves for patients in the high- and low-risk groups. **(D)** Forest plot of multivariate COX regression analysis combining clinical characteristics. **(E)** Risk factor triplot with risk score in the upper panel and survival outcome in the middle panel. The lower panel shows the molecular expression in the prognostic model. **(F)** Sankey plots between high- and low-risk groups with pyroptosis regulatory subgroups, pyroptosis differentially expressed gene subgroups, and survival status. *p < 0.05, **p < 0.01, and ***p < 0.001.

Using the LASSO algorithm, we analyzed the feasibility of constructing a CC model based on pyroptosis-related regulators. After 10-fold cross-validation, we obtained the best λ of 0.007903871 and 15 DEGs and their corresponding coefficients (**Figures 4A, B**). A prognostic model was constructed based on these eight genes (CXCL10, MMP12, CXCL13, MMP3, TNFAIP6, IDO1, CCL8, and KRT23), and the risk score was calculated. The samples were divided into high-risk and low-risk groups according to the median risk score values of 1163 CC samples, and their survival differences were compared. As shown in **Figure 4C**, the prognosis in the low-risk group was significantly better than that in the high-risk group. Subsequently, we identified independent prognostic factors of CC by univariable and multivariate COX regression analyses, as shown in **Figure 4D**; age, sex, stage, and high- and low-risk groups were all independent prognostic factors. Meanwhile, a risk factor plot shows that the higher the risk score, the worse the prognosis (**Figure 4E**). Combined with the results of the previous analyses, we plotted the relationship between risk score and pyroptosis regulatory subgroups, pyroptosis DEG subgroups, and survival status using a Sankey plot (**Figure 4F**). Based on the findings presented in **Figure 4F**, it is apparent that subjects from the disease group were initially categorized into two clusters (Cluster A (n=637); Cluster B (n=526)) based on pyroptosis-related gene expression, with subsequent differential gene analysis causing a decrease in the portion of subjects classified as Cluster B. The samples were then separated into high- and low-risk groups based on the median risk score. Notably, a higher proportion of patients who succumbed to the disease were found to be in the high-risk group, suggesting a link between the risk score and patient survival rates.

**Figure 5:**
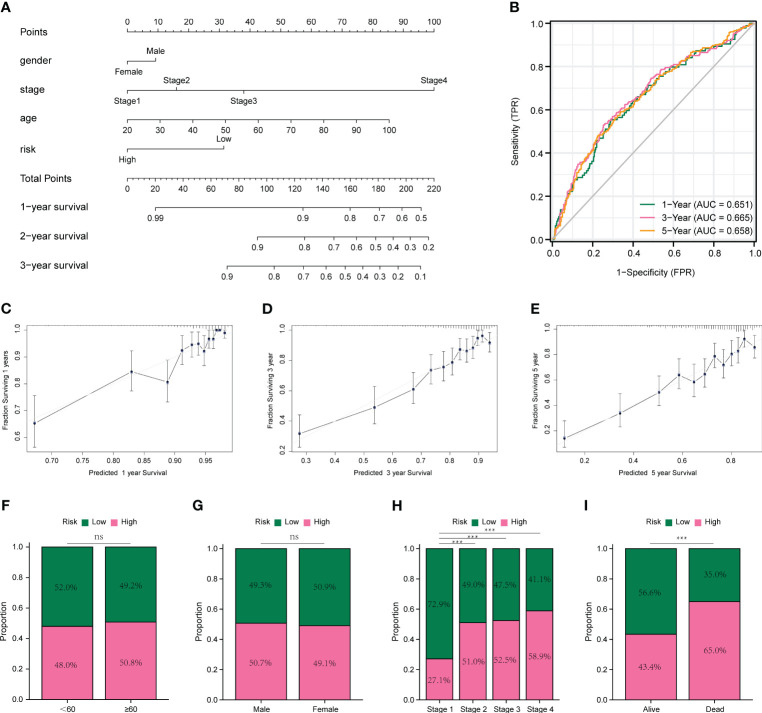
Prognostic models and evaluation of colon cancer. **(A)** Nomogram constructed based on multivariate COX regression results. **(B)** Time-dependent ROC curves for prognostic models. **(C-E)** Calibration curves for prognostic models at 1, 3, and 5 years. **(F)** Comparison of age in high- and low-risk groups. **(G)** Comparison of sex in high- and low-risk groups. **(H)** Comparison of clinical stage in high- and low-risk groups. **(I)** Comparison of high- and low-risk groups in survival status. *p < 0.05, **p < 0.01, and ***p < 0.001. ns means no significance.

We plotted a nomogram based on the results of multivariate COX regression, as shown in **Figure 5A**, and calculated a C-index of 0.738, which was evaluated by time-dependent ROC curves (**Figure 5B**) and correction curves at 1, 3, and 5 years (**Figures 5C–E**), which had a good prognostic assessment value. Subsequently, we analyzed the distribution of different clinical characteristics in the high- and low-risk groups and found no significant differences in the age or sex distribution in the high- and low-risk groups (**Figures 5F, G**). Significant differences in risk scores were observed amongst tumor stages. Specifically, stage 1 patients showed significantly lower risk scores compared to patients in stages 2, 3, and 4. Notably, patients in stages 2 and 3 exhibited lower risk scores than those in stage 4. These findings are illustrated in **Figure 5H**. Moreover, patients who succumbed to the disease had significantly higher risk scores than those who survived, as represented in **Figure 5I**.

### 3.5 Analysis of gene mutations and copy number variants

**Figure 6:**
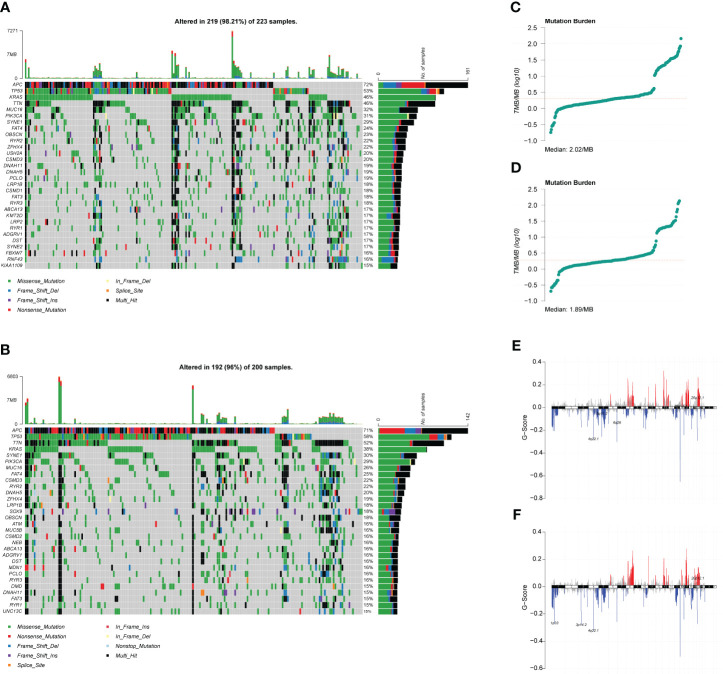
Mutation characteristics and copy number variation (CNV) analysis of high- and low-risk groups. **(A)** Waterfall plot of the top 30 mutated genes in the high-risk group. **(B)** Waterfall plot of the top 30 mutated genes in the low-risk group. **(C)** Tumor mutation burden (TMB) distribution characteristics of patients in the high-risk group. **(D)** TMB distribution characteristics of patients in the low-risk group. **(E)** CNV distribution characteristics of patients in the high-risk group. **(F)** CNV distribution characteristics of patients in the low-risk group.

The role of somatic mutations in cancer development and progression has been confirmed with the innovation of genetic testing technology, we analyzed the mutation characteristics of the high- and low-risk groups. First, we mapped the top 30 mutated genes in both groups (**Figures 6A, B**) using the maftools package. Subsequently, we analyzed the TMB of both groups and found that the median TMB of the high- and low-risk groups was 2.02/MB and 1.89/MB, respectively (**Figures 6C, D**). We then analyzed CNV in the high- and low-risk groups and found that CNV alterations occurred at multiple locations in both groups, but the overall differences between the two groups were not significant (**Figures 6E, F**). The term TMB refers to the number of non-synonymous genomic mutations observed in somatic cells within a specific genomic region. Typically, TMB is measured as the number of mutations per megabase (mut/Mb). Notably, TMB serves as an indirect marker for a tumor’s ability to generate novel antigens. This makes it a valuable predictor of the effectiveness of immunotherapeutic interventions, particularly for various cancer types. Tumors with higher TMB tend to harbor a significantly larger quantity of new antigens and, therefore, are more amenable to treatment with immunotherapy checkpoint inhibitors (ICIs). On the other hand, CNV, which stands for Copy Number Variation, is a genomic rearrangement event that generally results in the copy number increase or decrease of larger genome segments with a length exceeding 1 kb. CNV is primarily characterized by sub-microscopic deletions and duplications. It is crucial to note that both TMB and CNV serve as molecular indicators of genomic mutations. In cases where the TMB and CNV values of two groups exhibit no significant differences, it follows that there is little discrepancy between the groups concerning genomic mutations.

### 3.6 GSEA and GSVA for high- and low-risk groups

**Figure 7:**
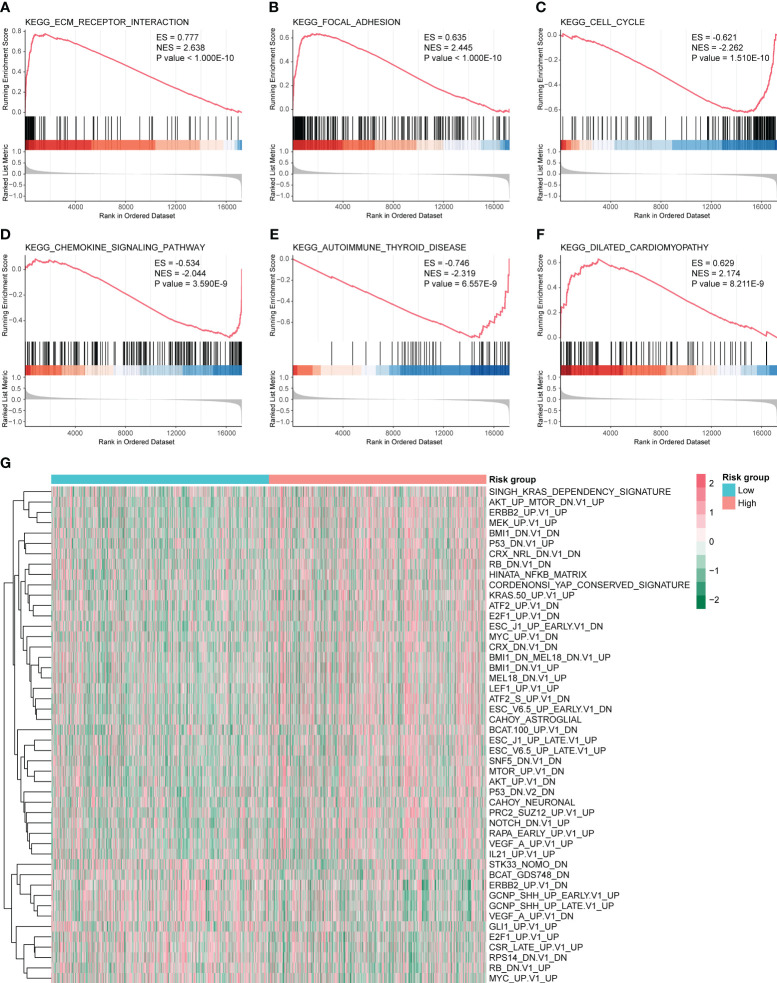
Gene set enrichment analysis (GSEA) and gene set variation analysis (GSVA) for high- and low-risk groups. GSEA pathways were significantly enriched in high- and low-risk groups, including **(A)** Kyoto Encyclopedia of Genes and Genomes (KEGG) ECM receptor interaction. **(B)** KEGG focal adhesion. **(C)** KEGG cell cycle analysis. **(D)** KEGG chemokine signaling pathway. **(E)** KEGG autoimmune thyroid disease. **(F)** KEGG dilated cardiomyopathy. ES and NES > 0 indicate that the pathway was enriched in the high-risk group, whereas ES and NES < 0 indicate that the pathway was enriched in the low-risk group. **(G)** Heat map of 48 pathways obtained from GSVA analysis.

Next, we performed GSEA and GSVA for the high- and low-risk groups of CC. As shown in **Figure 7A–F**, the high-risk group was mainly enriched in ECM receptor interaction, focal adhesion, dilated cardiomyopathy, and other pathways. The low-risk group functions were mainly enriched in the cell cycle, chemokine signaling pathway, and autoimmune thyroid disease pathways; detailed GSEA results are shown in **Table 2**. GSVA results show that the pathways were mainly enriched in 48 related oncogenic pathways, including the KRAS dependency signature, AKT up MTOR dn.v1 up, and ERBB2 up.v1 up (**Figure 7G**).

**Table 2:** GSEA enrichment analysis.

ID	enrichmentScore	NES	pvalue	p.adjust	qvalues
KEGG_ECM_RECEPTOR_INTERACTION	0.777053	2.637892	1.000E-10	9.300E-09	6.53E-09
KEGG_FOCAL_ADHESION	0.635028	2.444653	1.000E-10	9.300E-09	6.53E-09
KEGG_CELL_CYCLE	-0.62085	-2.26245	1.510E-10	9.362E-09	6.57E-09
KEGG_CHEMOKINE_SIGNALING_PATHWAY	-0.53368	-2.04398	3.590E-09	1.669E-07	1.17E-07
KEGG_AUTOIMMUNE_THYROID_DISEASE	-0.7459	-2.31859	6.557E-09	2.439E-07	1.71E-07
KEGG_DILATED_CARDIOMYOPATHY	0.629195	2.174141	8.211E-09	2.545E-07	1.79E-07
KEGG_ALLOGRAFT_REJECTION	-0.77253	-2.2584	2.066E-07	5.489E-06	3.85E-06
KEGG_HYPERTROPHIC_CARDIOMYOPATHY_HCM	0.615885	2.090769	2.552E-07	5.933E-06	4.16E-06
KEGG_DNA_REPLICATION	-0.74691	-2.2332	5.762E-07	1.191E-05	8.36E-06
KEGG_CYTOKINE_CYTOKINE_RECEPTOR_INTERACTION	-0.44139	-1.73063	1.346E-06	2.504E-05	1.76E-05
KEGG_PRIMARY_IMMUNODEFICIENCY	-0.72868	-2.15276	4.913E-06	7.808E-05	5.48E-05
KEGG_GLYCOSAMINOGLYCAN_BIOSYNTHESIS_CHONDROITIN_SULFATE	0.796482	2.082043	5.038E-06	7.808E-05	5.48E-05
KEGG_ARRHYTHMOGENIC_RIGHT_VENTRICULAR_CARDIOMYOPATHY_ARVC	0.582708	1.939575	8.733E-06	1.249E-04	8.77E-05
KEGG_INTESTINAL_IMMUNE_NETWORK_FOR_IGA_PRODUCTION	-0.6727	-2.08469	1.248E-05	1.659E-04	0.000116
KEGG_GRAFT_VERSUS_HOST_DISEASE	-0.71646	-2.09447	1.750E-05	2.170E-04	0.000152
KEGG_SPLICEOSOME	-0.49945	-1.81931	1.899E-05	2.208E-04	0.000155
KEGG_AXON_GUIDANCE	0.488584	1.777662	2.758E-05	3.017E-04	0.000212
KEGG_ASTHMA	-0.75614	-2.10676	3.252E-05	3.271E-04	0.00023
KEGG_TYPE_I_DIABETES_MELLITUS	-0.6705	-2.02195	3.342E-05	3.271E-04	0.00023
KEGG_PROTEASOME	-0.65492	-2.02253	4.062E-05	3.778E-04	0.000265
KEGG_T_CELL_RECEPTOR_SIGNALING_PATHWAY	-0.48859	-1.74726	1.340E-04	1.187E-03	0.000833
KEGG_REGULATION_OF_ACTIN_CYTOSKELETON	0.421614	1.629255	1.878E-04	1.588E-03	0.001114
KEGG_MISMATCH_REPAIR	-0.71249	-1.92015	2.335E-04	1.888E-03	0.001325
KEGG_CITRATE_CYCLE_TCA_CYCLE	-0.65747	-1.87918	3.351E-04	2.496E-03	0.001751
KEGG_HOMOLOGOUS_RECOMBINATION	-0.69362	-1.97812	3.354E-04	2.496E-03	0.001751
KEGG_CYTOSOLIC_DNA_SENSING_PATHWAY	-0.5857	-1.86538	4.172E-04	2.985E-03	0.002094
KEGG_COMPLEMENT_AND_COAGULATION_CASCADES	0.53016	1.726545	6.237E-04	4.296E-03	0.003015
KEGG_BASE_EXCISION_REPAIR	-0.64981	-1.89185	8.153E-04	5.416E-03	0.0038
KEGG_GAP_JUNCTION	0.478775	1.649082	8.678E-04	5.566E-03	0.003906
KEGG_PEROXISOME	-0.49368	-1.68601	1.634E-03	1.013E-02	0.007109
KEGG_NUCLEOTIDE_EXCISION_REPAIR	-0.5695	-1.76488	1.749E-03	1.032E-02	0.007239
KEGG_ANTIGEN_PROCESSING_AND_PRESENTATION	-0.50206	-1.68462	1.775E-03	1.032E-02	0.007239
KEGG_RNA_DEGRADATION	-0.5208	-1.6751	2.357E-03	1.328E-02	0.00932
KEGG_SYSTEMIC_LUPUS_ERYTHEMATOSUS	-0.43817	-1.5541	2.428E-03	1.328E-02	0.00932
KEGG_MAPK_SIGNALING_PATHWAY	0.350998	1.396499	3.562E-03	1.893E-02	0.013286
KEGG_AMINOACYL_TRNA_BIOSYNTHESIS	-0.54948	-1.68754	4.128E-03	2.122E-02	0.014892
KEGG_GLYCOSAMINOGLYCAN_DEGRADATION	0.664154	1.718671	4.221E-03	2.122E-02	0.014892
KEGG_HEMATOPOIETIC_CELL_LINEAGE	-0.45256	-1.54789	4.697E-03	2.299E-02	0.016135
KEGG_TIGHT_JUNCTION	0.40758	1.484808	5.760E-03	2.747E-02	0.019279
KEGG_ABC_TRANSPORTERS	-0.51438	-1.59405	8.211E-03	3.748E-02	0.026303
KEGG_UBIQUITIN_MEDIATED_PROTEOLYSIS	-0.39057	-1.4285	8.262E-03	3.748E-02	0.026303
KEGG_VASCULAR_SMOOTH_MUSCLE_CONTRACTION	0.409911	1.455422	8.555E-03	3.789E-02	0.026587
KEGG_PYRIMIDINE_METABOLISM	-0.42422	-1.47995	8.917E-03	3.857E-02	0.027067
KEGG_OOCYTE_MEIOSIS	-0.40633	-1.45508	9.666E-03	4.023E-02	0.028231
KEGG_NATURAL_KILLER_CELL_MEDIATED_CYTOTOXICITY	-0.40669	-1.48447	9.733E-03	4.023E-02	0.028231
KEGG_CALCIUM_SIGNALING_PATHWAY	0.356277	1.348768	1.117E-02	4.518E-02	0.031702

### 3.7 Differences in immune infiltration in the high- and low-risk groups

**Figure 8:**
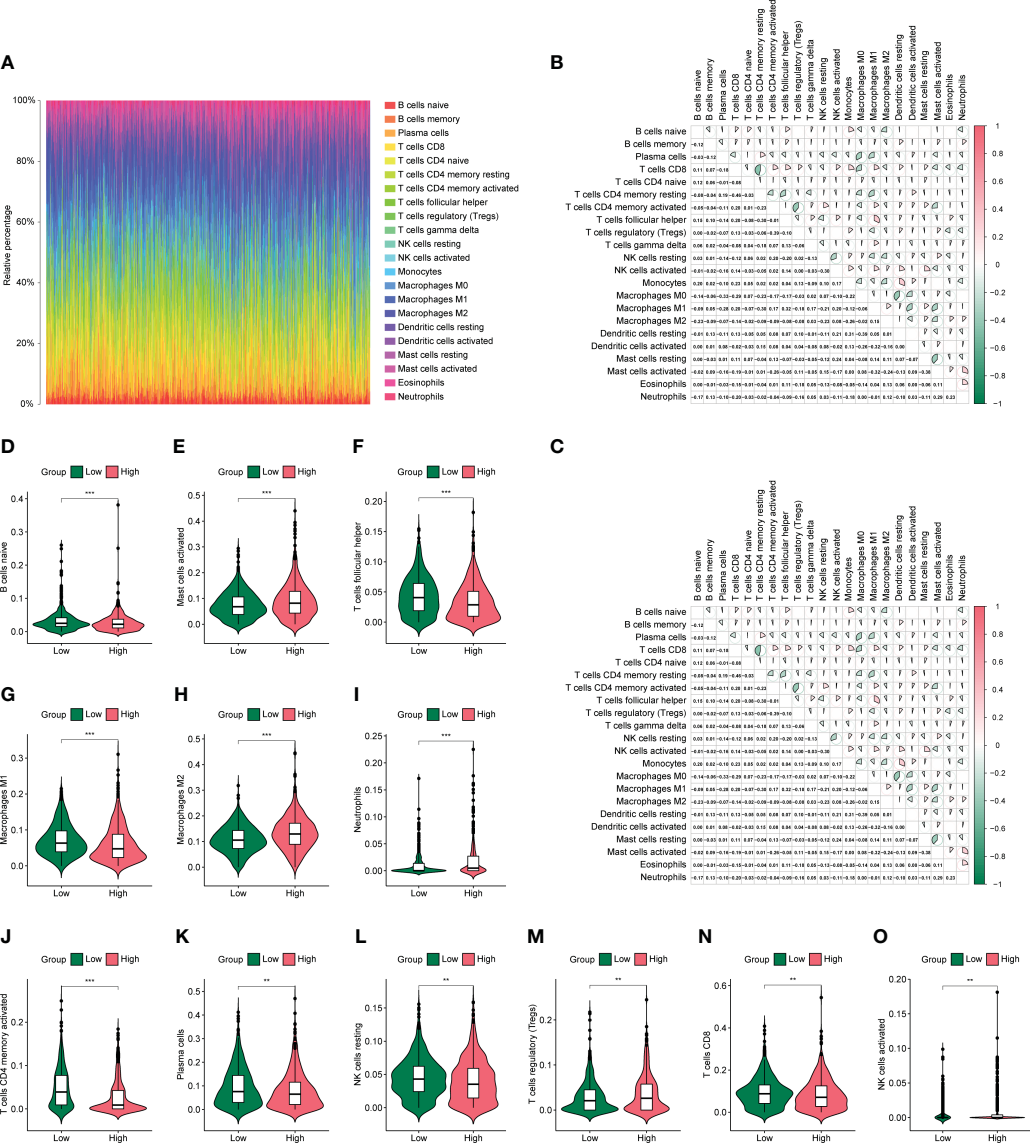
Differences in immune infiltration in high- and low-risk groups. **(A)** Panorama of 22 immune cell infiltrations calculated using the CIBERSORTX algorithm. **(B, C)** Heat map of association between 22 immune cells in the high- and low-risk groups. **(D-O)** Violin plot of differential numbers of 12 immune cells between high- and low-risk groups. *P<0.05, **P<0.01, ***P<0.001.

Based on previous results, in which the prognosis of the low-risk group was significantly better than that of the high-risk group, we speculate that there may be differences in immune infiltration between both groups. We determined changes in the level of immune cell infiltration in CC using the CIBERSORT algorithm, in which macrophage subpopulations account for a large proportion of infiltrating immune cells (**Figure 8A**). Correlation analysis shows correlations between the levels of infiltration of multiple immune cells, where red and green represent positive and negative correlations, respectively, with no significant differences between the high- and low-risk groups (**Figures 8B, C**). Immune cells were analyzed in both groups, and activated memory CD4+ T cells, follicular helper T cells, M1 macrophages, M2 macrophages, activated mast cells, and neutrophils were found to be different between the high- and low-risk groups (**Figures 8D–O**).

### 3.8 Immunotherapy and drug sensitivity analysis

**Figure 9:**
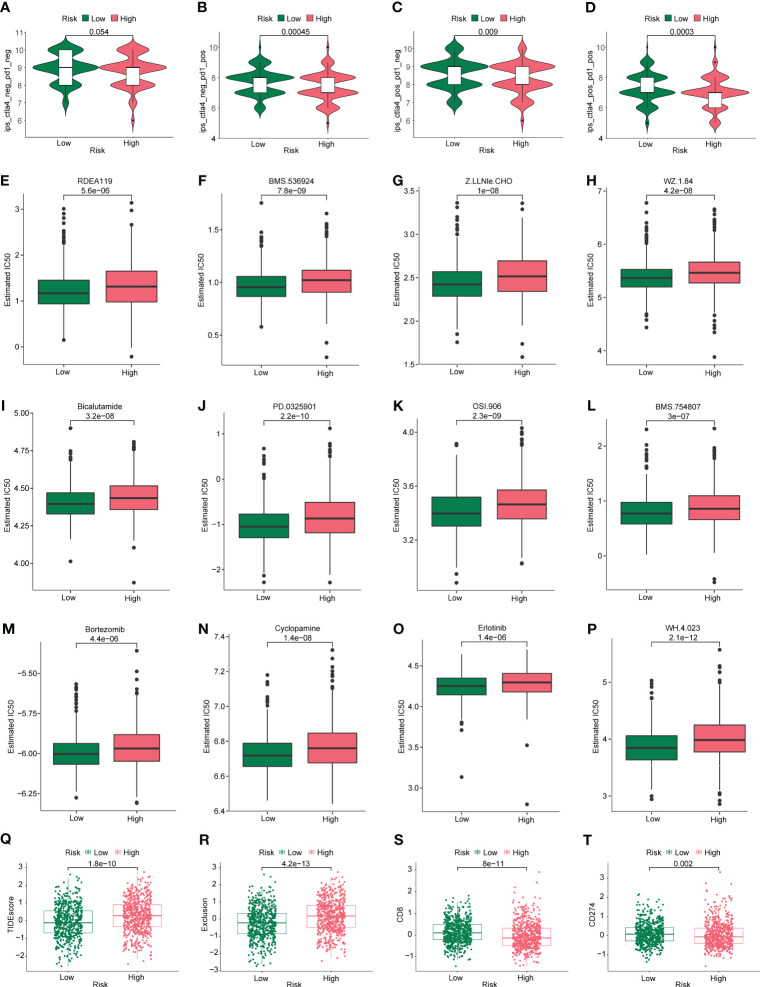
Immunotherapy and drug sensitivity analysis. Differences in **(A)** CTLA4 neg PD1 neg, **(B)** CTLA4 neg PD1 pos, **(C)** CTLA4 pos PD1 neg, and **(D)** CTLA4 pos PD1 pos between the high- and low-risk groups. Based on the GDSC database, differences in drug sensitivity of common antineoplastic drugs between the high- and low-risk groups were determined: **(E)** RDEA119, **(F)** BMS.536924, **(G)** Z.LLNle.CHO, **(H)** WZ.1.84, **(I)** bicalutamide, **(J)** PD.0325901, **(K)** OSI.906, **(L)** BMS.754807, **(M)** bortezomib, **(N)** cyclopamine, **(O)** erlotinib, and **(P)** WH.4.023. Differences in **(Q)** TIDE, **(R)** exclusive, **(S)** CD8, and **(T)** CD274 scores were calculated using the TIDE algorithm in the high- and low-risk groups.

First, we assessed the differences in IPS of patients in high- and low-risk groups based on TCIA database, as shown in **Figures 9A–D**. CTLA4 (–) PD1(+), CTLA4(+) PD1 (–), and CTLA4(+) PD1(+) were significantly different between high- and low-risk groups (P < 0.05). We then assessed the differences in sensitivity to common antitumor drugs between the high- and low-risk groups using the GDSC database. After analysis, we plotted the top 12 drugs with the most significant differences, such as bortezomib, erlotinib, cyclopamine, and bicalutamide (**Figures 9E–P**).

Given the important role that immunotherapy currently plays in tumors, we first assessed the sensitivity of patients in the high- and low-risk groups to immunotherapy using the TIDE algorithm. As shown in **Figures 9Q**, **R**, the TIDE scores were higher in the high-risk group than in the low-risk group, suggesting that immunotherapy responsiveness was better in the low-risk group than in the high-risk group. As shown in **Figures 9S, T**, the immune checkpoints CD8 and CD274 were scored for the tumors. The CD8 and CD274 scores were lower in the high-risk group than in the low-risk group, suggesting that they could be used as biomarkers, Undoubtedly, more mechanistic studies are needed to confirm this inference.

### 3.9 Validation analysis of GSE17537 dataset

**Figure 10:**
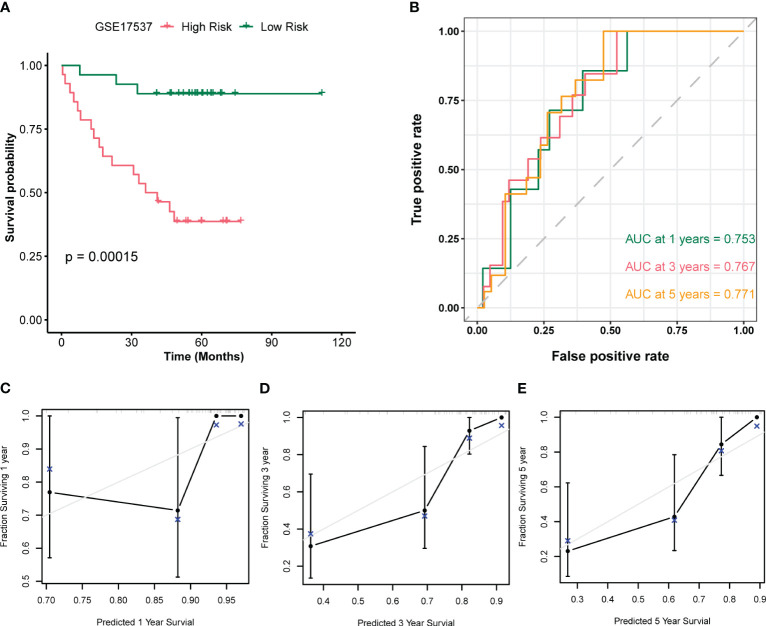
Validation analysis of the GSE17537 dataset. **(A)** Plot of survival curves for patients in the high and low risk groups of the GSE17537 dataset. **(B)** Time-dependent ROC curves for the prognostic model of the GSE17537 dataset. **(C-E)** Plots of 1-year **(C)**, 3-year **(D)**, and 5-year **(E)** calibration curves for the prognostic model.

In this study, we utilized multivariate Cox regression analysis of the merged dataset to calculate the risk scores of GSE17537 dataset using the same approach. We then separated the samples into high and low risk groups based on the median value of the risk scores, and constructed a prognostic model. After combining the Risk Score of the prognostic model with the prognosis survival information (OS.event) of GSE17537 dataset patients, we performed a prognostic KM curve analysis based on the Risk Score median value grouping (see **Figure 10A**). The results showed a significant statistical difference between the prognostic model risk score and GSE17537 dataset patients’ survival information (OS.event) (P < 0.001).

To further assess the accuracy of the prognostic model in predicting survival outcome (OS) of GSE17537 dataset patients, we plotted a time-dependent ROC curve which showed that the Risk Score of the prognostic model was moderately accurate in predicting survival outcome (AUC1 = 0.753, AUC3 = 0.767, AUC5 = 0.771, see **Figure 10B**). Moreover, we performed a 1-year (**Figure 10C**), 3-year (**Figure 10D**), and 5-year (**Figure 10E**) prognostic calibration analysis on the prognostic model combined with patient clinical information (age, gender, clinical stage), and plotted calibration curve graphs. The horizontal axis of the calibration curve graph represents the predicted survival probability of the model, and the vertical axis represents the actual data displayed survival probability. The lines and points of different colors represent the situation of model prediction at different time points. The closer the lines of different colors are to the gray ideal line, the better the prediction effect at that time point. From the graph, it can be seen that the calibration analysis of the prognostic model indicated that the 5-year and 3-year predictions were more reliable than the 1-year prediction.

### 3.10 Validation of protein expression levels of risk PRGs using IHC

**Figure 11:**
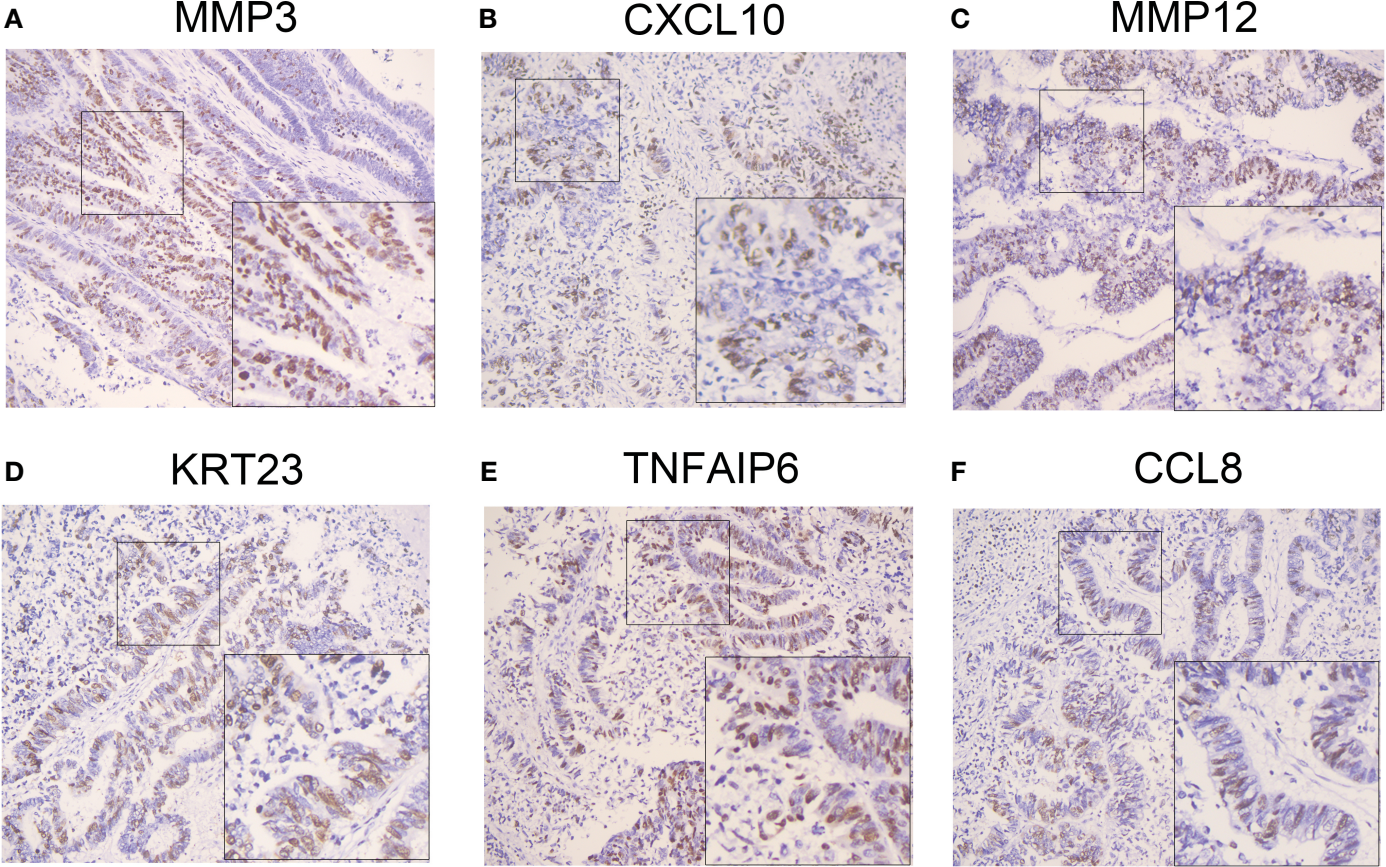
Validation of the selected genes in the prognostic model at the protein level. IHC staining identified the gene expression in clinical specimens. High expressions of MMP3 **(A)**, CXCL10 **(B)**, MMP12 **(C)**, KRT23 **(D)**, TNFAIP6 **(E)** and CCL8 **(F)** were presented in colon cancer tissues (n=25) by IHC staining. IHC: Immunohistochemistry. IHC: Immunohistochemistry. IHC stain, DAB, original magnification x 100 (inset, IHC stain, DAB, original magnification x 400).

We next evaluated MMP3,CXCL2, MMP12, KRT23, TNFAIP6 and CCL8 in CC tissues. The IHC staining results showed that all molecules were high expression (**Figure 11**).

## 4 Discussion

CC is one of the most common malignancies worldwide and the fifth-leading cause of cancer-related deaths in humans, posing a serious threat to human health (1). CC is occurring more frequently in younger patients and, despite improvements in early screening and treatment, the overall prognosis remains poor. Therefore, it is necessary to further investigate its pathogenesis and identify early prognostic factors and potential therapeutic targets (39). Recent innovations in genetic testing technology have provided conclusive evidence that mRNA levels and somatic mutations play key roles in cancer formation and progression, opening new opportunities for identifying novel biomarkers and developing therapeutic targets. Traditional predictive markers for CC prognosis, such as clinical parameters, MSI status, KRAS, and BRAF mutation status, have certain limitations. The construction of a polygene model is important for improving the accuracy of prognostic prediction and exploring new therapeutic targets.

Inflammation is closely associated with the development and progression of malignancies (40). Recently, a novel form of programmed cell death, pyroptosis, which is regulated by the GSDM protein family with a predominant inflammatory response, has been identified and is attracting increasing attention (9). Studies have shown that pyroptosis plays a dual role in inhibiting or promoting tumor development and is emerging as an attractive target in malignancy because of its significant role in the tumor immune microenvironment (TIME) and antitumor immunity (41, 42). Although several studies have explored the value of PRG signatures in predicting the prognosis and drug sensitivity of CC (12, 43), there is still a need for in-depth research to determine whether this new mode of pyroptosis gene signature can provide clinicians with therapeutic insights.

In this study, we selected 33 PRGs reported in the literature (20) and divided patients with CC into two subgroups (clusters A and B) through unsupervised cluster analysis based on the expression of these genes. PRG expression differed significantly between the two subgroups. To assess the differences between the modes of pyroptosis regulation, we first performed a differential analysis of the two subgroups and screened 59 DEGs. Further pathway enrichment analysis was performed to identify the biological functions of the PRGs. GO analysis shows that PRGs are closely related to biological processes such as receptor ligand activity, neutrophil chemotaxis, neutrophil migration, granulocyte chemotaxis, and cytokine receptor binding. KEGG analysis shows that PRGs affect pathways such as viral protein interaction with cytokines and cytokine receptors, rheumatoid arthritis, IL-17 signaling pathway, cytokine-cytokine receptor interaction, and chemokine signaling pathway. The above results corroborate the biological characteristics of pyroptosis, which influences the release of inflammatory factors, thus altering the TIME and regulating tumor progression (44).

To further clarify the impact of pyroptosis on the prognosis of patients with CC, we first selected 16 genes from the 59 PRGs that correlated with prognosis using univariate COX regression analysis. Finally, eight genes were screened using the LASSO regression and multivariate COX regression analyses. A prognostic model was constructed and risk scores were calculated based on these eight genes (CXCL10, MMP12, CXCL13, MMP3, TNFAIP6, IDO1, CCL8, and KRT23), and the samples were divided into high-risk and low-risk groups according to the median risk score values of 1163 CC samples.

TNFAIP6, a member of the hyaluronan-binding protein family, is a secretory protein containing a hyaluronan-binding domain. It is possibly involved in cell-cell and cell-matrix interactions during inflammation and tumorigenesis (45). Zhang et al. (46) and Cui et al. (47) found that TNFAIP6 promotes invasion and metastasis and indicates poor prognosis in patients with gastric cancer. Similarly, in our study, high TNFAIP6 expression was associated with a poor prognosis in CC.

MMP3 and MMP12 are both archetypal matrix metalloproteinases (MMPs), a group of protein hydrolases containing active Zn^2+^ that are involved in many functions related to self-stabilization, such as tissue repair and immune and pathological processes, including tumor, fibrosis, and infection (48). MMP3 is associated with tumor growth and metastasis in breast cancer (49) and CC (50). Similarly, Klupp et al. found that the level of MMP-12 protein expression in patients with CRC was significantly higher than that in healthy subjects and correlated with advanced CRC disease and vascular invasion (51). However, in our study, MMP3 and MMP13 were prognostic protective factors in patients with CC, and further studies are needed to explore the underlying mechanisms.

CXCL10 is a protein with a molecular weight of 8.7 kDa that belongs to the CXC chemokine family. The protein, one of the ligands of CXCR3, is important for stimulating T cell responses by inducing the expansion of CD4+ and CD8+ T cells and Th1 polarization, in addition to inducing chemotactic migration of immune cells. The CXCL10/CXCR3 axis has therapeutic potential by regulating angiogenesis, recruiting activated immune cells, and influencing the development of tumor cells, which in turn affects the TME (52). Thus, CXCL10 attracts effector lymphocytes to tumors and can be used as a therapeutic agent in CRC as well as in many cancer models (53). In contrast, another family member, CXCL13, is normally expressed in lymphoid organs and regulates the recruitment of B and antigen-presenting cells. CXCR5 is the primary receptor of CXCL13 and mediates chemokine function through specific downstream interactions (54). The CXCL13/CXCR5 axis is associated with tumor development, proliferation, and invasion. Aberrantly active CXCL13/CXCR5 signaling promotes cancer cell growth through complex molecular mechanisms in breast (55), intestinal (56), and lung (57) cancers. CCL8, also known as monocyte chemotactic protein 2 (MCP-2), is a small cytokine that belongs to the C-C chemokine family. CCL8 activates different immune cells, including mast cells, eosinophils, basophils involved in allergic reactions, monocytes, T cells, and NK cells associated with inflammatory responses (58). Several studies have confirmed that CCL8 not only promotes proliferation (59) and migration (60) but also enhances EMT and stemness of malignant tumors (61), which is consistent with our findings that CCL8 is a gene associated with poor prognosis in patients with CC.

IDO1 is a metabolic enzyme that regulates the levels of tryptophan and its metabolites *in vivo* by catalyzing the oxidative cleavage reaction of tryptophan and plays important biological functions in antibacterial, antitumor, immunomodulatory, and antioxidant activities (62). Many studies have shown that IDO1 is highly expressed in various cancers and is associated with tumor aggressiveness and poor prognosis (63, 64). IDO1 promotes an immunosuppressive environment by inhibiting T cell proliferation and stimulating Treg development. Inhibition of IDO1 reduces the number of immunosuppressive Tregs and restores cytotoxic T cell function. This may partly explain the difference in prognosis between the high- and low-risk groups.

The risk model constructed based on the expression of the above eight PRGs independently predicted the prognosis of patients with CC. Patients in the high-risk group had worse prognosis than those in the low-risk group. Next, we incorporated clinicopathological characteristics and risk scores into a multivariate COX regression model and constructed a nomogram. The best AUC was observed in the combined nomogram, in which the risk score had a promising prognostic signature. Further validation of this prediction model in ROC curves and calibration plots revealed that the risk score had equally good efficiency in predicting longer survival in patients with CC. Subsequently, we analyzed the distribution of different clinical characteristics in the high- and low-risk groups and found that there was no significant difference in the distribution of age or sex, whereas in the tumor stage, the later the TNM stage, the higher the risk. In the death group, the proportion of high-risk patients was significantly higher than that in the low-risk group.

To explore the underlying molecular mechanisms of the risk signature, we performed GSEA and GSVA. The results show that the high-risk group was mainly involved in ECM receptor interaction and focal adhesion, whereas the low-risk group was mainly involved in the cell cycle, chemokine signaling pathway, and cytokine receptor interaction. These enrichment results indicate that the risk signature was strongly related to inflammation-related pathways, which is consistent with the biological role of pyroptosis.

Tumors exist in a complex immune microenvironment (65). As a type of inflammatory cell death, pyroptosis significantly affects the TIME and thus regulates tumorigenesis and progression (66). By analyzing the differences in immune cells between the high- and low-risk groups, we found that the low-risk group had high levels of naïve B cells, follicular helper T cells, M1 macrophages, memory activated CD4+ T cells, plasma cells, resting NK cells, and CD8+ T cells, whereas the high-risk group had more activated mast cells, M2 macrophages, neutrophils, regulatory T cells (Tregs), and activated NK cell. This may explain the prognostic differences between the high- and low-risk groups related to the effect of prognostic models being constructed with PRGs in the immune microenvironment. The TIME plays a key role in the immunosuppression of cancer, which leads to tumorigenesis, progression, and insensitivity to immunotherapy and chemotherapy. We further explored whether a predictive model constructed based on PRGs could distinguish patient sensitivity to immunotherapy or chemotherapy. First, we found that the TIDE scores were significantly higher in the high-risk group than in the low-risk group, suggesting that immunotherapy responsiveness was better in the low-risk group than in the high-risk group. In addition, the exclusive score, which usually reflects the strength of immune escape, was higher in the high-risk group than that in the low-risk group, suggesting that immunotherapy may be less effective in the high-risk group. Further analysis of the immune checkpoint CD8 and CD274 scores of tumors revealed that CD8 and CD274 scores were significantly lower in the high-risk group than in the low-risk group, suggesting that they could act as biomarkers. Similarly, we assessed the difference in IPS of patients in the high- and low-risk groups using the TCIA database and found that CTLA4(+) PD1(+) was significantly higher in the low-risk group than in the high-risk group (P < 0.05).

Pyroptosis, a type of programmed cell death characterized by an inflammatory response, can significantly influence the TIME and thus regulate the development and prognosis of malignancy (67). Although some relevant studies have been reported (12, 43, 68), the exploration of prognostic models based on PRGs in CC remains necessary and valuable. Among them, Chen et al. (68) performed the analysis based on the samples in TCGA, and the GEO data set was the validation set. Compared to this study, our research differs significantly in three main aspects. Firstly, our selection of data sets is broader. While the study solely focused on colon adenocarcinoma (COAD) and relied on the TCGA database as the only data source, potentially introducing sample bias, we expanded our scope to include datasets from both the TCGA and GEO databases to ensure a more representative sample. Secondly, we used a different analysis workflow. While the prior study employed supervised clustering as their primary analysis method, we began with unsupervised clustering analyses on the combined dataset (TCGA+GEO) of colon cancer samples, thereby identifying two subtypes. We subsequently conducted additional analyses based on these subtypes and validated our results using a supplementary GSE17537 dataset. Thirdly, we conducted different wet experiments to validate our findings. In contrast to the previous study, which validated pyroptosis-related genes through the HPA database and qPCR, we performed immunohistochemistry experiments on clinical samples to evaluate the expression of pyroptosis-related genes. In contrast to the reported studies, we performed the first combined analysis of multiple datasets from TCGA and GEO databases (a total of 1163 CC samples), which has better innovation and value (**Table S1**). Unlike the above-mentioned studies, we not only performed immune infiltration analysis for different risk groups but also compared the mutation characteristics and susceptibility to common antitumor drugs in both groups and finally screened and identified eight novel pyroptosis-related prognostic genes. Based on the risk score, the samples were effectively divided into high-risk and low-risk groups, and high-risk patients were found to have a significant immunosuppressive microenvironment and a poorer effect on immunotherapy, which partly explains the poorer prognosis of high-risk patients. Previous studies have confirmed that the immunosuppressive microenvironment is a key factor for worse prognosis in patients with CC (69); therefore, the construction and comprehensive analysis of this prognostic model provides a new marker for the treatment and prognosis of patients with CC.

However, our study has some limitations. First, this prognostic model is based on retrospective datasets, so more prospective evaluation is needed to validate the accuracy of the model. Second, although IHC has been performed to validate the different protein expression levels of risk DRGs, the role and mechanism of pyroptosis in the immune microenvironment of CC requires further investigation.

## 5 Conclusion

In summary, we comprehensively analyzed the landscape of PRGs in patients with CC and constructed an 8-gene prognostic model based on pyroptosis-related prognostic genes, which effectively classified patients into high-risk and low-risk groups. Pathway enrichment analysis of the high- and low-risk groups revealed that they were mainly enriched in inflammatory response-related pathways. Compared to the low-risk group, patients in the high-risk group had worse OS, an immunosuppressive microenvironment, and lower sensitivity to immunotherapy and drug treatment. In conclusion, the comprehensive analysis of PRGs in this study helps to predict prognosis and guide individualized and precise treatment for patients with CC.

## Data availability statement

The original contributions presented in the study are included in the article/**Supplementary Material**. Further inquiries can be directed to the corresponding authors.

## Ethics statement

The studies involving human participants were reviewed and approved by Institutional Ethics Committee of Panyu Maternal and Child Care Service Centre of Guangzhou (He Xian Memorial Affiliated Hospital of Southern Medical University). The patients/participants provided their written informed consent to participate in this study.

## Author contributions

MW: Conceptualization, Methodology, Writing-Original Draft. SH: Conceptualization, Methodology, Software. XW: Formal analysis. SS: Resources. SD: Resources. SZ: Writing-Review & Editing. RY: Supervision, Funding acquisition. HD: Supervision, Funding acquisition. All authors contributed to the article and approved the submitted version.

## Supplementary material

Supplementary Figure 1 Boxplot graphs of TCGA+GEO merged dataset before and after batch effect removal. **(A)** Boxplot graph of TCGA+GEO dataset before batch effect removal. **(B)** Boxplot graph of TCGA+GEO dataset after batch effect removal. **(C, D)** PCA graphs of merged dataset before **(C)** and after **(D)** batch effect removal.

Supplementary Figure 2 Signal pathway diagram. **(A)** Cytokine-cytokine receptor interaction pathway. **(B)** Viral protein interaction with cytokine and cytokine receptor pathway.

SUPPLEMENTARY TABLE 1 Patient demographic.
