# Supplementary figures and images for: A pyroptosis-related gene signature that predicts immune infiltration and prognosis in colon cancer
Source: Front Oncol. 2023 Jul 12;13:1173181. doi: 10.3389/fonc.2023.1173181 (PMC10369052; doi:10.3389/fonc.2023.1173181)

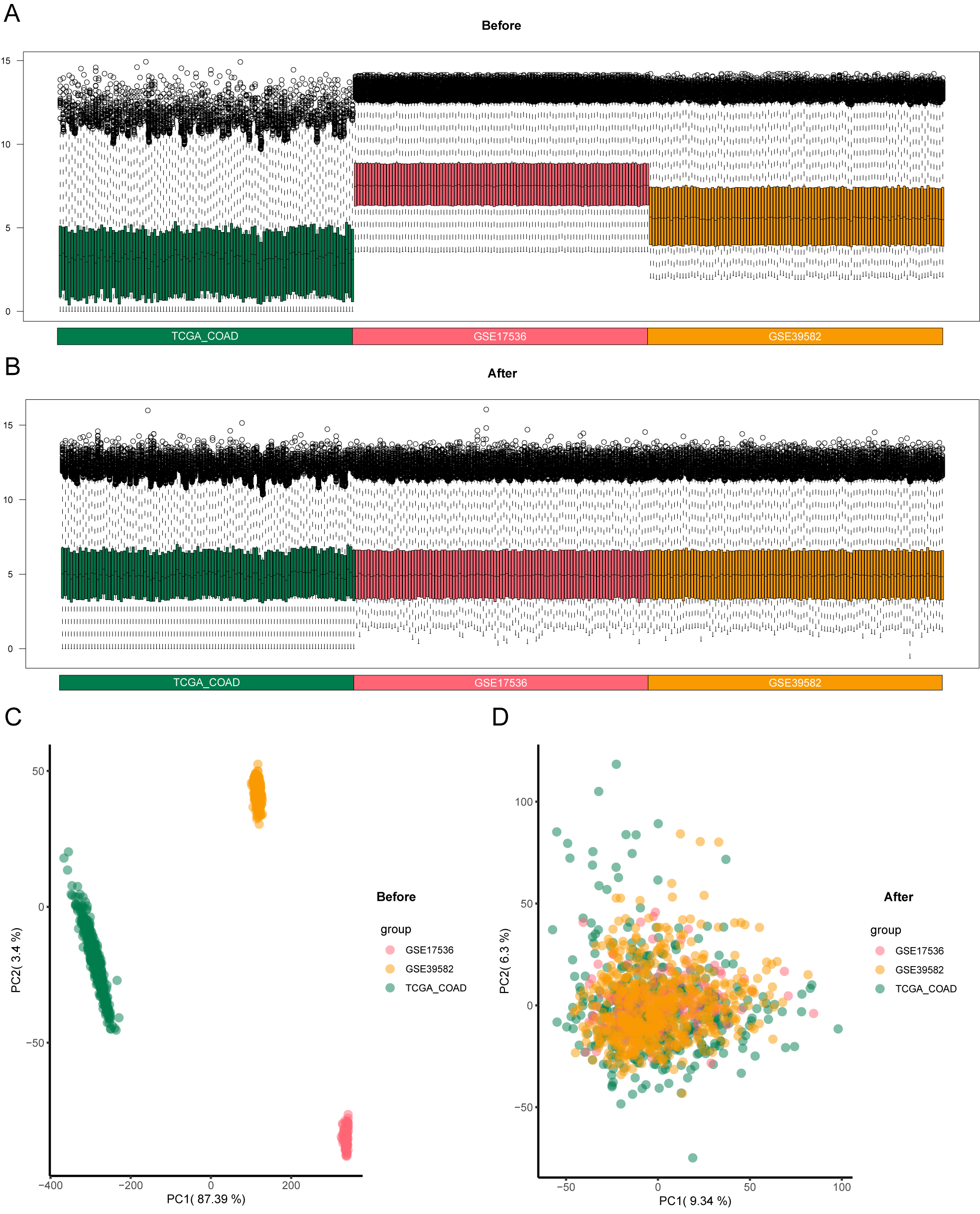

Supplement: Supplementary Figure 1 — Boxplot graphs of TCGA+GEO merged dataset before and after batch effect removal. (A) Boxplot graph of TCGA+GEO dataset before batch effect removal. (B) Boxplot graph of TCGA+GEO dataset after batch effect removal. (C, D) PCA graphs of merged dataset before (C) and after (D) batch effect removal. [file Image_1.tif]

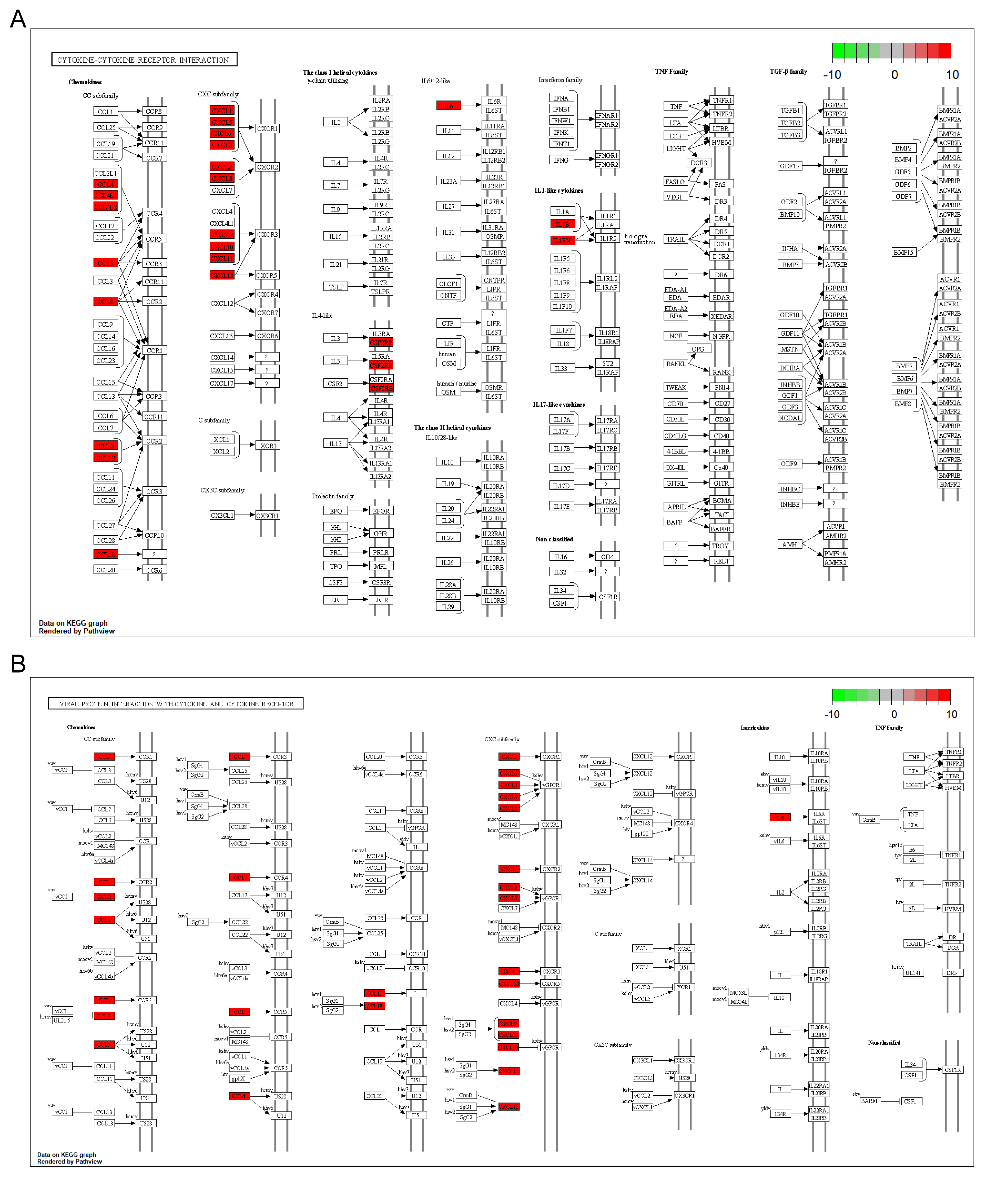

Supplement: Supplementary Figure 2 — Signal pathway diagram. (A) Cytokine-cytokine receptor interaction pathway. (B) Viral protein interaction with cytokine and cytokine receptor pathway. [file Image_2.tif]
