# Supplementary material for: A pyroptosis-related gene signature that predicts immune infiltration and prognosis in colon cancer
Source: Front Oncol. 2023 Jul 12;13:1173181. doi: 10.3389/fonc.2023.1173181 (PMC10369052; doi:10.3389/fonc.2023.1173181)
Supplement: SUPPLEMENTARY TABLE 1 — Patient demographic. [file Table_1.docx]

**Table S1**. Patient demographic.

|  | **TCGA (n=430)** | **GSE17536 (n=177)** | **GSE39582 (n=556)** | **Total (n=1163)** |
| --- | --- | --- | --- | --- |
| **Status** |  |  |  |  |
| Alive | 336 | 104 | 369 | 809 |
| Dead | 94 | 73 | 187 | 354 |
| Overall  Survival Time (Year/Median) | 1.840 (0.003-11.699) | 3.523 (0.077-11.879) | 4.333 (0.083-16.75) | 2.984 (0.003-16.75) |
| Age (Year) | 68.6 (31.2-90.1) | 66 (26-92) | 68 (22-97) | 68.05 (22-97) |
| Gender |  |  |  |  |
| Male | 232 | 96 | 307 | 635 |
| Female | 198 | 81 | 249 | 528 |
| Stage |  |  |  |  |
| I | 73 | 24 | 32 | 129 |
| II | 165 | 57 | 258 | 480 |
| III | 121 | 57 | 203 | 381 |
| IV | 60 | 39 | 59 | 158 |
| Unknown | 11 |  | 4 | 15 |
